# The Use of a Comprehensive Concept of Capability for Wellbeing Assessment: A Best-Fit Framework Synthesis

**DOI:** 10.1007/s10728-024-00488-w

**Published:** 2024-08-19

**Authors:** Jasper Ubels, Karla Hernandez-Villafuerte, Erica Niebauer, Michael Schlander

**Affiliations:** 1 Division of Health Economics, German Cancer Research Center (DKFZ), Im Neuenheimer Feld 280, 69120 Heidelberg, Germany; 2 Mannheim Medical Faculty, University of Heidelberg, Mannheim, Germany; 3 Alfred‐Weber Institute, University of Heidelberg, Heidelberg, Germany

**Keywords:** Health economics, Quality of life, Outcome measurement, Patient-reported outcomes, Capability approach, Best-fit framework synthesis

## Abstract

Developing an instrument with the capability approach can be challenging, since the capability concept of Sen is ambiguous concerning the burdens that people experience whilst achieving their capabilities. A solution is to develop instruments with a comprehensive concept of capability, such as the concept of ‘option-freedom’. This study aims to develop a theoretical framework for instrument development with the concept of option-freedom. A best-fit framework synthesis was conducted with seven qualitative papers by one researcher. Two researchers supported the synthesis by discussing interim results during the synthesis. A priori concepts of option-freedom were used to deductively code against. Themes and subthemes were developed inductively when data did not match a priori themes. Seven paper were identified that fulfilled the eligibility criteria. Four themes emerged from the synthesis. (1) Option Wellbeing represents a range of options that need to be satisfied for individuals to experience wellbeing. (2) Self-Realization represents that there are experiences in an individual’s life that have value beyond realizing options. (3) Perceived Access to Options represents the perceived ability of individuals to realize freedoms. (4) Perceived Control represents the experience of having control. Developing an instrument with the proposed framework has two benefits. First, it acknowledges the importance of assessing impediments in realizing capabilities for wellbeing assessment. Secondly, the themes form a broad informational base by including themes related to subjective wellbeing. Future research should study the feasibility of implementing the framework for instrument development.

## Introduction

Health technology assessment is the practice of assessing the value of new health technologies to inform decision-making [1, 2]. Some jurisdictions use the Quality Adjusted Life Year (QALY) to assess the value of a health technology. The QALY is a measure that combines both information about health-related quality of life and length of life, by adjusting a life year with the quality of that life year. This quality adjustment is calculated by performing weighted adjustments to scores from instruments that measure changes in health-related quality of life. The weights that are used for these adjustments reflect the utility of living [3, 4].

However, it is argued that the impact of health technologies is not limited to improving the health of an individual. Therefore, some scholars argue for the use of the capability approach as a framework to assess the value of health technologies [4, 5]. They claim that value should be assessed on a broad informational base that goes beyond health-related quality and length of life, and takes into consideration what is important for the people themselves.

In this context, the capability approach has emerged as an alternative framework for the development of instruments. Two key concepts in the capability approach are functioning and capability. Functionings reflect those things that an individual can do or be, for instance, to be nourished or to be a respected member of the community. Capabilities of individuals represent the combination of opportunities that an individual ‘can do’ or ‘can be’ [6].

This conceptualization of the capability approach was developed by Sen [7], who originally understood capability as a form of positive freedom [8]. This is also reflected in definitions of capability by Sen that have been used in instrument development, where capability instruments have been focusing on what individuals can do or can be in different dimensions. However, in terms of operationalizing the approach in the form of instruments that can be used to assess wellbeing, this definition has two limitations. The first limitation is that this definition is relatively narrow [8]. The burdens that people might experience while achieving these capabilities are not well reflected in this definition, which affects the degree to which researchers that aim to apply these concepts in instrument development recognize these burdens [9]. The second limitation is that understanding a capability as those things that an individual can do or can be is relatively vague, which poses a further challenge for operationalizing the capability approach. In the context of wellbeing assessment with self-reported instruments, this results in difficulties in identifying whether certain elements of wellbeing are more appropriately assessed in terms of capabilities or other constructs, such as functionings [9]. It should be noted that these limitations are not a critique or a commentary on the concept of capability by Sen itself, but rather on the limitations of the definitions of Sen have been used to operationalize the approach into instruments that can be used to assess wellbeing in the field of health and health economics.

One solution for these limitations is to develop an instrument with an a priori definition of capability that is clearer and more comprehensive. In the context of our study, ‘a priori concepts’ mean the implicit or explicit concepts that developers have when they interpret qualitative data, for example in the case of a best-fit framework synthesis or the development of a new instrument. Robeyns [8] has proposed such a concept. She argues that a capability is best understood as an ‘option freedom’, which is a concept developed by Pettit [10]. A more detailed explanation of the concept of option freedom can be found in the appendix.

We would like to stress that we do not aim to engage in a conceptual discussion on the clearest definition of capability in this paper, or what kind of freedom a capability exactly is. Rather, the aim of this paper is to show how alternative definitions of capability could support the development of instruments. In this context, conceptualizing capability as an option freedom in the context of instrument development has two advantages. First, the concept of option freedom stresses that a capability can be considered as a freedom that can blocked or burdened. As such, by assessing wellbeing based on this definition, more attention is paid to the blocks and burdens that people might experience while achieving their capabilities [9]. Second, operationalizing a clearer definition of capability might facilitate the distinction between aspects of wellbeing that represent capability, and aspects that represent other elements of wellbeing that might be relevant for its assessment besides capability.

The concept of option freedom has not been operationalized yet for measurement. This study aims to develop a theoretical framework based on the concept of ‘option of freedom’, which can be used to develop an instrument that assesses the wellbeing of individuals.

## Methods

In this study, a best-fit framework synthesis is conducted [11]. With this method, a theoretical framework is identified a priori, which is used to code the data against. Data that do not fit the a priori theoretical model are reinterpreted with thematic analysis techniques. The result of such an analysis is a new or further refined conceptual framework (see Table 1).

**Table 1:** Themes, subthemes and quotes from the participants in the qualitative research paper

Themes	Subthemes	Description	Quotes
Option wellbeing	Physical wellbeing	Physical wellbeing represents the physical health related wellbeing of an individual, such as the absence of discomfort or pain	*“I just wouldn’t want to be in pain all the time”. (Female, 72 years, PC), Sutton and Coast *[21] *“I’m nearly 86, I’ve got all me faculties thank goodness, I saw my husband and he never ever knew me at times.” (Female, aged 85), Grewal, Lewis *[20]
	Emotional wellbeing	Emotional wellbeing represents the affective wellbeing of an individual. Examples are feeling happy, or the absence of sadness or nervousness	*“It’s contentment, I think, really—satisfaction. I love the theatre, I love the cinema … if it’s just to go off for a day somewhere and have a meal in a pub… And I think that’s very essential, it’s just the simple pleasures of life really” (male, aged 69), Al-Janabi, N Flynn *[19] *“…obviously it’s [mother’s illness] been hard, it’s been upsetting…and visiting her now isn’t exactly a barrelful of laughs… I guess it’s saddening …” (Female, 29), Al-Janabi, N Flynn *[19]
	Social wellbeing	Social wellbeing represents the wellbeing associated with having good social contacts with, for example, friends or family	*“One should take good care of the kids and the entire family, so that everyone is healthy and they can work properly and prosper.” Greco, Skordis-Worrall *[22] *“- you just feel so isolated” (Male, Employed), *[17]
	Environmental wellbeing	Environmental wellbeing is related to the wellbeing associated with feeling settled in the house and the wider neighborhood	*“A house should have a toilet, a bathing shelter, there should be a rubbish pit, and the house should be well taken care of. Even if you have all these things but they are not put to good use, diseases will be there.” Greco, Skordis-Worrall *[22] *“I get a lot of pleasure out of sitting in the garden now… I suppose we are lucky living where we are… “(female, aged 73), *[20]
	Activity wellbeing	Activity wellbeing represents the wellbeing derived from being able to do things for fun, or for relaxation	*“Work is important. Just to go out and do things that aren’t mind numbing if you know what I mean.” (F employed), Kinghorn, Robinson *[17]
Reflective wellbeing	Having a role	Having a role represents wellbeing derived from the ability to do things that provide a sense of worth and is linked to the identity of individuals	*“I do like playing…competitive sport…it’s got a bit of an edge …. I suppose through that there’s a bit of an achievement thing and it’s quite nice to be in a team or to be a captain for one of the teams” (Male, 29), Al-Janabi, N Flynn *[19] *I’ve got four grandchildren, and I can’t pick none of them up, in fact, if they jump on me, I fall over. (Male, Not employed), Kinghorn, Robinson *[17] *It brings a feeling of inner satisfaction really to think you have been of some use… Especially with bereavement, (male, aged 69), Grewal, Lewis *[20]
	Having dignity	Having dignity is related to the wellbeing associated with being a respected member of a community, as well as being able to conduct as a being of worth	*“A person who changes clothes is seen as living a good life. She changes dirty clothes after a bath, and puts on clean ones, and looks good. When she is amongst people, she is not shy. As for me, I may have to wash the few I have to put on when I go in public.” Greco, Skordis-Worrall *[22] *I think some people get the wrong idea when they get these people who’ve got this Alzheimer’s that they wanna be directed and pushed – they don’t want that do they? (Female, 74 years, GP), Sutton and Coast *[21]
	Being independent	Being independent represents the wellbeing derived from individuals being able to access options, without being interfered or having to rely on others. It is also linked to the perception of individuals to have a sense of agency, even though of problems that might rise up	*“I would like to drive a bit more. Because I’m losing my independence. I have to rely on my husband to take me shopping now.” (Female, Retired), Kinghorn, Robinson *[17] *“A person should be independent because when sick she doesn’t wait for someone to tell her what to do, men at times neglect that you are struggling.” Greco, Skordis-Worrall *[22] *‘My family found it hard to believe their dad had Alzheimer’s… I said, ‘‘You heard what he called me a little while ago—‘Make me a cup of coffee, Nurse’’’. I said, ‘‘How do you think that makes me feel?’’ to my children.’ (female, aged 85), Grewal, Lewis *[20]
	Self-determination	Self-determination represents the wellbeing that is a consequence of being able to make those choices that are meaningful to individuals	*[Regarding the choice of conducting NIPT] “I just really think that women should be given ownership of the information and they can decide what they want to do.” Kibel and Vanstone *[18] *“As a Physics Teacher, to do 6 years without any promotion is pretty unusual really because they’re in such short supply. And I was beginning to feel left on the shelf.” [Male, 28], Al-Janabi, N Flynn *[19]
Perceived access to options	Access due to physical wellbeing	Depending on the state of physical health, individuals have varying levels of access to options	*[The chest infection] “just made it miserable for a week or two, I couldn’t get out or about…” [Male, 75], Al-Janabi, N Flynn *[19] *“The negative thing with diabetes is when people at work ask if you can join them for something after work. No, I can’t, I’m going home to take my injection and have dinner. You get a little tied up, you know.” (#17; Woman, 60 years old, Type 2 DM), Engström, Leksell *[23] *“Because of the arthritis and that, I can’t work, so there’s… work mates, you know, no Friday night when you’ve got the wages and you can enjoy it, a couple of beers. There’s none of that.” (Male, Not employed), Kinghorn, Robinson *[17]
	Access due to emotional wellbeing	Also the emotional state of individuals can influence the access to options. People who do not feel well can experience difficulties accessing options	*“I started getting depression… it’s like yesterday, I didn’t have a wash, I didn’t have a shave, I didn’t get up, I didn’t even unlock the door, and that was it” (Male, not employed, A), Kinghorn, Robinson *[17] *“…if I feel fine up top I’ll have a better day. But if I wake up in the morning and I don’t feel right up here, you know the day is going to be worse “ (Male, not employed, B), Kinghorn, Robinson *[17]
	Access due to social wellbeing	The social context of individuals also has an influence on the access of options of an individual. For example, having a wide support network eases the access the options	*When I came out of hospital he [husband] done everything I mean, he cooked the food and he’s never cooked in his life [laugh] … And all the washing, ironing he did. (Female, 72 years, PC), Sutton and Coast *[21] *“[Good life means] someone who when you meet on the way, can help you.” *[22] *“I have many close friends and acquaintances who support me, which means a lot. Above all, in tough periods when it’s difficult to manage my blood glucose levels and so on, it’s a great support for me.” (#28; Man, 31 years old, Type 1 DM), Engström, Leksell *[23]
	Access due to environmental wellbeing	The environment of an individual, inside and outside the house, also has an influence on the ease of access to options. An appropriate environment can facilitate the access to options	*“I went in for this flat because it’s wheelchair friendly … I’m hoping that I’d lay here in a box, because it was a very deliberate act of me to look for somewhere where I can be independent for as long as possible.” (Female, 67 years, GP), Sutton and Coast *[21] *It was lovely down here at one time, but it’s just frightening now… I wouldn’t walk when it were dusk from here to the top of the hill… because there’s weirdos on the canal. (female, aged 66), Grewal, Lewis *[20]
	Access due to activity wellbeing	The activities of an individual influences the access to options. Due to an individual doing things, more options will come along the way	*“Because of the arthritis and that, I can’t work, so there’s… work mates, you know, no Friday night when you’ve got the wages and you can enjoy it, a couple of beers. There’s none of that.” (Male, Not employed), Kinghorn, Robinson *[17] *“At ante-natal classes …six of us really gelled and just became the closest of friends. It was like we’d known each other for years and years and years. … we see each other all of the time and we help each other out which is great.“ (Female, 32), Al-Janabi, N Flynn *[19]
	Access due to financial resources	The financial situation of an individual has an influence on the ability to access options. The more financial resources an individuals has, the easier it is for an individual to access options	*[Regarding the choice to do a NIPT] “If I had to pay for it, I would borrow from my friends or relatives. But I would just do anything possible to avoid a miscarriage.” Kibel and Vanstone *[18] *“I’m reasonably fortunate… in so far as that we’ve got the two pensions… we’re able to go off… We grabbed a cheapie flight at the end of April… flew down to Nice…” (male, aged 70), Grewal, Lewis *[20]
	Access due to technologies	The access to technologies gives the individuals the opportunity to access other options. For example, new drugs or therapies can take away limitations which are a consequence of disease	*“I think the insulin pump is fantastic. Because it gives me freedom.” (#24; Woman, 64 years old, Type 1 DM), Engström, Leksell *[23] *“if there was no NIPT, I’d be left with a positive result from my Integrated Prenatal Screening, not willing to do an amniocentesis because I’m not willing to you know, go with that risk, so the rest of my pregnancy I have that added stress of thinking that I have a high chance of having you know, a baby with Down Syndrome”, Kibel and Vanstone *[18]
Perceived control	Management	Management represents the perceived ability of individuals to manage the burdens or limitations in access to options. Being able to manage these burdens and limitations is conductive to the wellbeing of an individual	*“This morning, I got up—5 o’clock—I took my first pain killers, went back to bed again so that I was ready to get up to have my shower at half past six, or else, by the time you start taking them they haven’t taken effect and you’re trying to move around. So, yeah, you’ve got to think ahead…” (Female, not employed, A), Kinghorn, Robinson *[17] *[Regarding the management of diabetes] “It’s not easy, it’s an endless struggle to try to maintain good blood glucose levels. (…) It’s like walking a line.” (#24; Woman, 64 years old, Type 1 DM), Engström, Leksell *[23]
	Evaluation	Evaluation represents the assessment by an individual between his or her realized options and the preferred level of option realization	*“It is a constant sadness, that I’ve lost my sight… (…) But it’s nothing I get hung up on in my everyday life. (…) I consider myself as having a good quality of life.” (#1; Man, 49 years old, Type 1 DM), Engström, Leksell *[23] *[Regarding NIPT] “I was going to go for the blood work but then I just was like, like you know what, I would rather not think about it, I would rather not stress about it.”, Kibel and Vanstone *[18]

Our best-fit framework synthesis followed three general steps: (1) the identification of an a priori framework or theory; (2) the development of a search strategy to identify studies; and (3) the data analysis and synthesis of a new or updated framework. The enhancing transparency in reporting the synthesis of qualitative research statement was used as a checklist (see Table 2) to ensure the current study’s transparency [12].

**Table 2:** ENTREQ checklist

No	Item	Page were item is addressed
1	Aim	5
2	Synthesis methodology	5
3	Approach to searching	6
4	Inclusion criteria	6
5	Data sources	6
6	Electronic search strategy	Addressed in article by Ubels et al. [9]
7	Study screening methods	Addressed in article by Ubels et al. [9]
8	Study characteristics	Addressed in article by Ubels et al. [9]
9	Study selection results	Addressed in article by Ubels et al. [9]
10	Rationale for appraisal	Page 6
11	Appraisal items	Page 6
12	Appraisal process	Page 6
13	Appraisal results	Pages 7–8
14	Data extraction	Page 6
15	Software	Page 6
16	Number of reviewers	Page 7
17	Coding	Page 7
18	Study comparison	Page 8
19	Derivation of themes	Page 7
20	Quotations	Page 7–14
21	Synthesis output	Page 7–14

### Identification of an a Priori Framework

A definition of capability is used as an a priori ‘lens’ for analysis. As mentioned, Sen’s definition is ambiguous concerning the burdens that people might experience whilst achieving their capabilities [8, 9]. Therefore, capability is defined as an option freedom [10]. The advantage of the definition of option freedom over other definitions of capability is, that freedom is understood as something that cannot only be “externally” blocked (through, for example, laws that limit capabilities), but also be “internally” blocked (through, for example, societal conditioning of women to not follow education, which results in women themselves not wanting to follow education). There are also alternative ways to define capability, however, to evaluate the advantages and disadvantages of these would entail a philosophical debate that is beyond the scope of this paper. For a more in-depth discussion about the various concepts of freedom and how they relate to capability, see Robeyns [8].

### Search Strategy

The papers included in this synthesis are selected from an earlier study where we conducted a literature review [9]. The literature review included articles that explain how instruments were developed to assess capabilities in the context of wellbeing assessment in the field of health (these articles are hereafter called ‘development papers’). The instruments and associated development papers were identified with a comprehensive pearl-growing search strategy [13]. Further details about the search strategy can be found in Ubels et al. [9].

For the present analysis, development papers were considered eligible when they contained ‘rich’ qualitative data. Articles containing rich qualitative data are those articles that do not only mention the themes measured by the instrument, but also explain how these themes were developed. These themes are then supported with quotes from the participants whose insights were used to develop the themes (hereafter called the participants).

The quality of the identified studies was appraised by JU using the Consolidated Criteria for Reporting Qualitative Research (COREQ) checklist [14]. These criteria were not used as a standard to exclude studies, given the discussion around the exclusion of qualitative studies in literature reviews [15]. Rather, the checklist was used by the author to ensure that no important criteria had been missed in the reading of the studies which might have influenced the development of the framework. A further post-hoc sensitivity analysis was conducted to evaluate if excluding studies with missing or unclear information could have influenced the result of synthesis. This was done by comparing the themes identified in the development papers that provided a complete report according to the COREQ checklist, and the development papers that missed reporting some aspects [15, 16].

### Data Synthesis

A best-fit framework synthesis was conducted to develop themes that can be used for instrument development. First, data was analyzed by extracting the complete result sections of the development papers to Excel. Second, two a priori themes, ‘Options’ and ‘Access to Options’, were used to deductively analyze the data from the development papers sequentially. These a priori themes are based on the concept of Option Freedom [10]. Data that did not fit the a priori themes, were inductively analyzed using thematic synthesis methods [11]. Out of the inductively analyzed data, new codes and new themes were identified. After defining new codes and themes, the data were again analyzed and coded against the newly developed codes and themes. This process was iterative, to further define the themes as well as the coherence between the themes. Coding was conducted line by line. The illustrating quotations from the development papers are all from participants of the respective studies.

The synthesis was primarily conducted by the principal author, JU. JU has a background in health sciences and health economics. During the coding process, the analysis was discussed with EN, who provided a critical external view on the coding process and framework in development. EN is an anthropologist by training and has experience in conducting qualitative research in a variety of different settings. The results were discussed with KHV, who provided a critical view on the interpretation of the data and presentation of the results. KHV is an experienced health economist with a broad background in both the theoretical aspects of health economics and economical evaluations.

## Results

Seven out of the twelve development papers identified by Ubels et al. [9] were eligible for inclusion in the best-fit framework synthesis [18–23]. These seven papers form the basis of the content of (1) a capability instrument that is developed to assess wellbeing in individuals affected by chronic pain, developed by Kinghorn et al. [17], (2) a non-invasive prenatal testing related capability wellbeing questionnaire, developed by Kibel and Vanstone [18], (3) the ICECAP-A (ICEpop CAPability measure for Adults) developed by Al-Janabi et al. [19], (4) the ICECAP-O (ICEpop CAPability measure for Older people) developed by Grewal et al. [20], (5) the ICECAP-SCM (ICECAP Supportive Care Measure) developed by Sutton et al. [21], (6) the women’s capability index developed by Greco et al. [22] and (7) a diabetes specific instrument for measuring patient reported outcomes and experiences in the Swedish national diabetes register developed by Engström et al. [23]. The other four papers included in that review, which concern the development of four other instruments [24–28], did not contain the rich data necessary for a best-fit framework synthesis at the time of writing this article and were therefore excluded from the current study.

Based on the COREQ checklist, some observations can be made. Generally, the studies contained detailed information about the data analysis and the findings. However, the relationship between the interviewers and the participants was unclear in two papers [20, 22]. Furthermore, in some papers, information about the personal characteristics of the researchers was missing [17, 19, 20, 22]. It was thus difficult to assess how the individual backgrounds of the researchers could have potentially influenced the interpretation of the data. A detailed review of the qualitative papers included in the present study can be found in Ubels et al. [9].

### Development of the Model

**Fig. 1:**
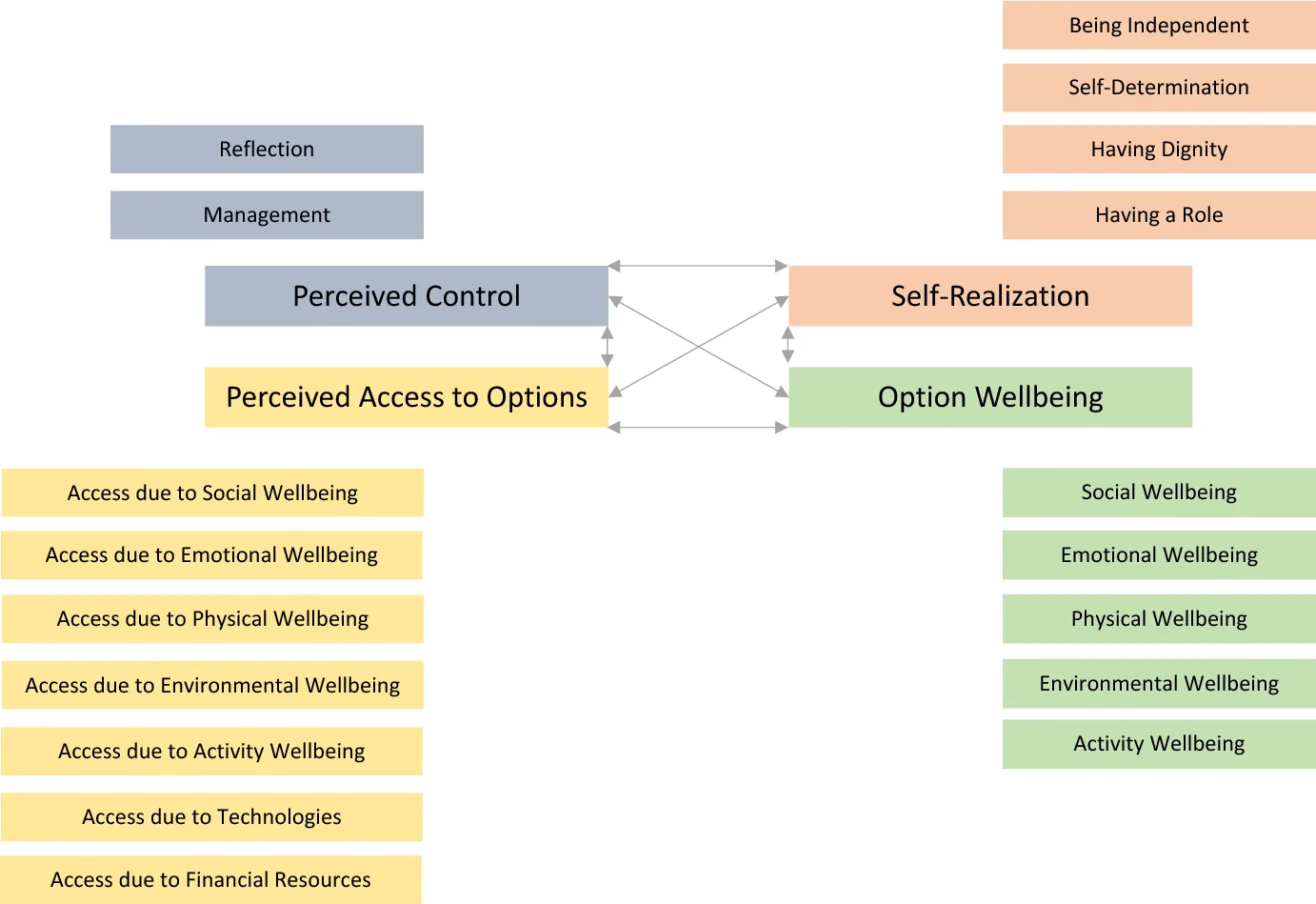
Graphical representation of themes and subthemes

Four main themes emerged as a result of the synthesis: (1) Option Wellbeing, (2) Self-Realization, (3) Perceived Access to Options, and (4) Perceived Control. The next sub-sections will describe each theme with their related subthemes. Table 1 shows an overview of the themes and subthemes, with definitions and associated quotes. Figure 1 presents the four main themes and their associated subthemes that are identified in this study.

#### Option Wellbeing

The theme Option Wellbeing represents the importance for people to have options that are beneficial in terms of happiness or satisfaction. This theme is essentially based on the a priori concept of Options and reflects that in order for an individual to experience happiness or life satisfaction. If these options cannot be realized to an adequate level, the happiness or satisfaction of individuals is negatively affected. These options could be subdivided into five abstract subthemes: (1.1) Physical Wellbeing, (1.2) Emotional Wellbeing, (1.3) Social Wellbeing, (1.4) Environmental Wellbeing, and (1.5) Activity Wellbeing. A definition of each of these subthemes can be found in Table 1.

Depending on the personal characteristics of the participants of the studies, different subthemes appear to be more important in the development papers. For instance, the relevance of achieving a perceived adequate level of Physical Wellbeing had a predominant role when the themes were developed with participants affected by disease [17, 21, 23], with elderly [20], as well as with participants groups for which specific medical technologies are developed [18].

The following quote highlights the negative impact of a lack of Physical Wellbeing:

*I’m in pain 24-7, whether I’m laying down, in the bath or hanging from the lightshade, I’m in bloody pain (Male, not employed, B), Kinghorn, Robinson *[17].

The importance of Environmental Wellbeing and Activity Wellbeing particularly emerged from four development papers [17, 19, 20, 22]. The remaining subthemes, Emotional Wellbeing, and Social Wellbeing, were mentioned to be important by the researchers in all the development papers [17–23].

#### Self-Realization

The theme Self-Realization rests on the idea that there are aspects of an individual’s life that have value beyond options that generate happiness or satisfaction. These aspects could be divided into subthemes (2.1) Having a Role, (2.2) Having Dignity, (2.3) Being Independent, and (2.4) Self-Determination. Pursuing these subthemes might even come at a cost of happiness or satisfaction. The importance of being able to experience these aspects was noted in several development papers [17–22].

Having a Role involves more than simply doing certain things for pleasure or satisfaction, as would be the case in the subtheme Activity Wellbeing. Having a Role refers to being able to do those things that provide a sense of worth and identity [17, 19, 20, 22]. For example, Kinghorn, Robinson [17] noted that some men perceived themselves to be less masculine since they could not carry heavy things to help their partners.

*…you feel inadequate. Well I do, when my missus starts…unloading the car, and I walk into the house and sit down. (M employed, A), Kinghorn, Robinson *[17].

Having Dignity represents the importance of the perceived social standing of an individual in their community. The subtheme is related to the ability of individuals to conduct themselves as beings of worth and be respected by other members of their community. In this context, several papers mentioned the importance of recognition by other people [17–19, 21–23], as well as the ability of people to take care of matters which are considered to be private [21, 22].

*I’ve got my self-respect, she [carer] doesn’t stand there if I’m having a shower and all that, she just makes sure the windows are covered … we all want our self-respect no matter who we are. (Female, 68 years, PC), Sutton and Coast *[21].

Being Independent is related to participants being free to make their own choices, without being influenced by limitations and having to rely on others to access options. Being Independent can also be understood as the more fundamental perception of individuals to have agency over their own lives, especially in troubled times. This subtheme was particularly highlighted in five papers [17, 19–22].

The subtheme Self-Determination refers to the ability to make valuable choices related to options that are meaningful for the individual. Terminology used by the authors included: (1) the freedom to express oneself without being oppressed [22], (2) the ability of doing certain actions without asking for consent [22], (3) the ability of individuals to achieve goals or move forward in their lives [19] and (4) being able to make choices about aspects that influence their lives [18, 21].

*I’m staying here until I get carried away. I’ve worked hard and paid for it, and this is my abode and I’m quite happy with it. (Male, 72 years, GP) Sutton and Coast *[21].

The need to choose, however, was also considered to be overwhelming in some circumstances [18, 21]. For instance, when the difficulty of processing the information required to understand and/or weight alternatives overloaded the capacity of individuals to deal with this information, choices were reported to be extremely difficult.

*[Concerning non-invasive prenatal testing (NIPT)] Well I think a lot of women don’t really want to think about it but I think they need to understand what the possible outcomes are. Like I think everybody knows that Down’s syndrome is trisomy 21 but there seems to be a lot of vagueness and even a lot of confusion about the testing itself and about what they’re actually looking for. Kibel and Vanstone *[18].

#### cPerceived Access to Options

As mentioned, the best-fit framework synthesis started with two a priori concepts, one of them being Access to Options. During the synthesis, the a priori concept Access to Options was re-conceptualized into the theme Perceived Access to Options. This change stresses the subjective experienced nature of access to options that were captured by the development papers. The theme Perceived Access to Options represents the perceived ability to access options that are of value to an individual’s wellbeing. It reflects individuals’ perceptions regarding barriers that exist in their lives.

The subthemes under Option Wellbeing play an instrumental role here (e.g. physical wellbeing, emotional wellbeing, etc.), since they affect an individual’s perceived ability to access various options: Additionally, the Perceived Access to Options is also influenced by two other factors, the financial situation of participants and the use of (medical) technologies [17–20, 22, 23]. A short description per subtheme in the context of access to options can be found in Table 1.

The subthemes here are mutually interdependent, meaning that burdens or blocks in the access to one option could influence the access to other options. For example, lower levels of Physical Wellbeing might limit the access to options of the subtheme Social Wellbeing, as can be observed in the following quote.

*It’s sad not daring to go [on a trip]. (...) Since it [hypo- glycaemia] is a threat, it feels like a lower quality of life. (...) You get a little scared of exposing yourself to situations other than what you are used to. (#17; Woman, 60 years old, Type 2 DM) Engström, Leksell *[23].

#### Perceived Control

The theme Perceived Control represents the importance of people having a perceived ‘grasp’ over their lives. Authors noted in their development papers the importance of this perception of control for individuals [17–20, 22]. This perception of control is defined by its subthemes Management and Evaluation.

The subtheme Management represents the perceived ability of individuals to use particular options to reduce the effects of factors that limit access to other options. The subtheme Management represents the strategic activity of individuals to deal with limitations. Individuals aim to achieve those options that have the most value to them, despite the potential restrictions in access to other options [17, 18, 21–23].

*This morning, I got up—5 o’clock—I took my first pain killers, went back to bed again so that I was ready to get up to have my shower at half past six, or else, by the time you start taking them they haven’t taken effect and you’re trying to move around. So, yeah, you’ve got to think ahead… (Female, not employed, A), Kinghorn, Robinson *[17].

Evaluation is a more fundamental theme. It represents the assessment of the realized options of an individual compared to his/her preferred options. This evaluation is influenced by the ability of individuals to access those preferred options. Moreover, the evaluation also is influenced by individuals’ expectations regarding the future ability to access those options [17, 19–21, 23]. When the burden to access an option is extremely high, adaptation of preferences might occur. For example, a participant adapted her preferred options in the context of Activity Wellbeing, because she could not walk anymore.

*That would be a good point to put next to the hobbies, er walking…. I’m not able to do that, so then I had to look for something else to occupy my mind. So right, then—I always did do a lot of knitting and cross stitch—I take a lot of interest in that, and reading. (Female, Retired), Kinghorn, Robinson *[17].

Some researchers suggest that an important prerequisite for adaptation is acceptance of the limitation to access various options [17, 23].

## Relationship Between Themes

It should be noted that all the themes and subthemes interact with each other, which is signified arrows between the themes in Fig. 1. To illustrate, limitations in the Perceived Access to Options affected the themes Option Wellbeing and Self-Realization when the participants were unable to have Perceived Control [17, 22, 23]. This particularly affected the Emotional Wellbeing of individuals [18–20].

*…my health broke down again … which came as a shock… I had to give up work immediately …and it cast a long shadow because it’s always there in the background, you never know when it might jump on you. So you live with uncertainty. (Female, 78), Al-Janabi, N Flynn *[19].

*I mean my discomfort is emotional rather than physical and I have days when I feel really good, and don’t worry, but if I have a very down period … I mean nobody wants to experience pain but I’m quite sure these days they can do things to relieve you of pain, but it’s just the emotional thing really which is more, especially when you’ve got nobody to talk it through with … (Female, 83 years, PC) Sutton and Coast *[21].

Vice-versa, if individuals experienced Option Wellbeing and had a sense of Self-Realization, they were also experiencing Perceived Control and did not experience limitations in their Perceived Access to Options [23]. For instance, some participants considered themselves to be well off, even though limitations or burdens in access existed due to chronic disease [17].

### Post-Hoc sensitivity analysis

Regarding the COREQ checklist [14], four development papers did not provide information on all items [17, 19, 20, 22]. The exclusion of these papers did not result in less or different themes emerging from the data, since all the themes and subthemes were identified in the three remaining papers. Nevertheless, excluding these four papers might have had an impact on the depth and transferability of the theoretical model.

## Discussion

Pettit’s theory [10] proved to be a useful a priori ‘lens’ to identify the differences between options, how these options are accessed, and how certain elements limit access to valued options. The application of the framework to the qualitative data meant that the a-priori themes were re-conceptualized. Furthermore, due to its clarity, the application of Pettit’s theory also supported the re-interpretation of the qualitative data of the included studies. Although the included studies were developed to identify valuable capabilities, the actual analysis of the qualitative data with the a priori concepts related to option freedom led to the identification of elements that are important for wellbeing, but do not directly reflect freedom itself. Rather, participants in the studies described other concepts to be important for their wellbeing that more closely reflected a sense of control and experienced wellbeing, and therefore did not fit the a priori themes related to option freedom.

Our analysis suggests another theme that might have a key role in wellbeing assessment: the theme Perceived Control. The theme Perceived Control shows similarities with the concept of dis-capability of Bellanca, Biggeri [29]. They suggest that an individual can be considered dis-capable when he or she is unable to manage the limitations that are imposed on her or his life due to, for example, a disability.

Two subthemes are linked to the theme Perceived Control, Management and Evaluation. With respect to Management, previous empirical studies have already showed the importance of disease management for the wellbeing of the individuals. Gibbins, Bhatia [30] studied preferences of advanced cancer patients with chronic pain regarding pain management. They found that the effect of pain and the side effects of medication on patients’ independence, understood as the range of choice and control patients have over their lives, is a stronger influence on the patients’ subjective wellbeing than reducing experienced pain itself. Other studies show similar results concerning the importance of controlling the limitations that might be caused by disease [31], not only in respect to pain management [32, 33], but also in respect to controlling the symptoms of mental health problems [34]. The second subtheme Evaluation reflects the idea that individuals reevaluate the value of their options in relation to the limitations in access to those options. This mechanism is known as adaptation [31, 35, 36]. In the context of patient wellbeing, facilitating adaptation to chronic disease can be seen as an integral part of treatment, particularly when no other possibilities to reduce symptoms exist [37].

Two further themes that emerged are Self-Realization and Option Wellbeing. The two themes are closely related to the way that subjective wellbeing is conceptualized [38]. The subthemes related to the themes Self-Realization and Option Wellbeing show similarities to common abstract elements of wellbeing observed in different fields of study. Qizilbash [39] argues that one reason for these similarities across different fields of studies is that on a fundamental level, these different lists of elements reflect commonly shared values. It is therefore not surprising that the themes and subthemes developed in this study reflect elements of wellbeing that have been found to be important in other research fields.

An overall comparison of the themes of our synthesis with the frameworks from the qualitative studies shows that the themes and subthemes of our framework are generally broader and more abstract than the themes and subthemes of frameworks that have been created to develop a disease or health technology related capability instrument [17, 18, 21, 23]. This also means that some context-specific themes and subthemes might be missing, such as themes that are specifically important for managing diabetes in Sweden [23]. Our framework also includes more detailed subthemes related to how disease might affect achieving capabilities and how these limitations are managed than frameworks that have been developed with relatively healthy populations [19, 20, 22]. An important caveat here is that our framework is not developed with direct involvement of members of the public and thus lacks legitimacy compared to the qualitative frameworks that were included in our synthesis, since they have been developed with members of the public.

On a conceptual level, the argument to include functionings related to subjective experiences as valuable outcomes in wellbeing assessment could be considered controversial in the context of the capability approach. One of the arguments for assessing wellbeing in terms of capabilities instead of functionings that represent the subjective experiences of individuals, is that individual might report experiencing higher levels of wellbeing than expected. This is because they adapt to adverse circumstances, such as ill-health [36]. Additionally, it has been argued that the evaluative space of capabilities covers functionings, such that the assessment of capabilities is sufficient for the assessment of wellbeing. To illustrate, finding that an individual has the capability to be well nourished is sufficient to argue that an individual is well-off in this respect, irrespective of the actual level of nourishment of that individual.

However, when developing capability approach based instruments, it might be beneficial to combine the measurement of perceived capabilities and the subjective experience of living with this capabilities. One finding that emerged from our analysis is that the subjective experiences of individuals did not always seem to be related to their perceived capabilities. This did not seem to be necessarily choice based (i.e. individuals could experience wellbeing but actively choosing against it to pursue other valuable functionings), but seem to rather be a result of an implicit or explicit evaluation of their capabilities. Therefore, wellbeing could be assessed more comprehensively with self-report instruments when both capabilities and the subjective experience of living with those capabilities are evaluated. This is in line with Clark [40], who argues that the informational base of the capability approach should be expanded to include a wider range of subjective experiences. These experiences could be seen as a form of functioning. Thus, the proposed framework provides a broad informational base on which to assess wellbeing.

In the context of assessing the value of new health technologies, the present framework has several advantages over using Sen’s definition of capability for the development of instruments. First, it stresses that people who can access options *with* difficulty cannot be considered to have an equal level of capability compared to people who can achieve the same options *without* difficulty. The assessment of these burdens is crucial to understand individuals’ wellbeing (see also Cookson [41] for a discussion in the context of the QALY). Second, the framework includes the assessment of how people experience their capabilities in terms of the perceived control and its effect on the subjective wellbeing of individuals. This could additionally also result in a more comprehensive evaluation of health interventions. Some interventions do not aim to expand capabilities by improving health, but rather support individuals adapting to limitations imposed by health due to, for example, chronic disease [37].

## Limitations

The proposed framework is based on the synthesis of a limited number of qualitative papers that have mainly conducted research in high-income countries, with a particular focus on Europe. Furthermore, the synthesis depends on the interpretation of data by a select number of researchers. Because of these limitations, it is unclear how generalizable the framework is beyond this setting. Still, the themes seem to reflect common abstract elements that are important for wellbeing, given the parallels between the themes identified in this study and lists created by other authors.

## Appendix

Access to Options.

Different takes exist on the nature of these blocks and burdens. When an option exists and can be accessed, then this combination counts as an option freedom. Pettit defines three distinct ways in which access to an option can be blocked for an individual: objectively, subjectively or both objectively and subjectively. An objective block to an option can be a lack of medication. For instance, the option to be free of symptoms of an individual affected by asthma is blocked in such a case. A subjective block might occur when an individual has psychological barriers to access healthcare, For instance, an individual affected by depression might be too ashamed to go to therapy. In this case, the block is subjective, as there is nothing objectively stopping the individual to seek for medical help.

Besides the possibility of access being blocked, Pettit also states the possibility of access being burdened. This means that an option is still accessible, but with difficulties. For example, there might be high costs involved in receiving medical help, which could mean that an individual has to work overtime. As such, the access to the option to get medical support is not blocked, but burdened.
